# An uncommon and insidious presentation of renal cell carcinoma with tumor extending into the inferior vena cava and right atrium: a case report

**DOI:** 10.1186/s13256-016-0888-5

**Published:** 2016-05-03

**Authors:** Hou Tee Lu, Jen Lim Chong, Norliza Othman, Simon Vendargon, Shamsuddin Omar

**Affiliations:** 1 Clinical School Johor Bahru, Jeffrey Cheah School of Medicine and Health Sciences, Monash University Malaysia, 8 Jalan Masjid Abu Bakar, 80100 Johor Bahru, Johor Malaysia; 2 Department of Cardiology, Sultanah Aminah Hospital, Jalan Abu Bakar, 80100 Johor Bahru, Johor Malaysia; 3 Department of Radiology, Sultanah Aminah Hospital, Jalan Abu Bakar, 80100 Johor Bahru, Johor Malaysia; 4 Department of Cardiothoracic Surgery, Sultanah Aminah Hospital, Jalan Abu Bakar, 80100 Johor Bahru, Johor Malaysia; 5 Department of Urology, Sultanah Aminah Hospital, Jalan Abu Bakar, 80100 Johor Bahru, Johor Malaysia

**Keywords:** Right atrial mass, Renal cell carcinoma, Thrombus

## Abstract

**Background:**

Renal cell carcinoma is a potentially lethal cancer with aggressive behavior and it tends to metastasize. Renal cell carcinoma involves the inferior vena cava in approximately 15 % of cases and it rarely extends into the right atrium. A majority of renal cell carcinoma are detected as incidental findings on imaging studies obtained for unrelated reasons. At presentation, nearly 25 % of patients either have distant metastases or significant local-regional disease with no symptoms that can be attributed to renal cell carcinoma.

**Case presentation:**

A 64-year-old Indian male with a past history of coronary artery bypass graft surgery, congestive heart failure, and diabetes mellitus complained of worsening shortness of breath for 2 weeks. Incidentally, a transthoracic echocardiography showed a “thumb-like” mass in his right atrium extending into his right ventricle through the tricuspid valve with each systole. Abdomen magnetic resonance imaging revealed a heterogenous lobulated mass in the upper and mid-pole of his right kidney with a tumor extending into his inferior vena cava and right atrium, consistent with our diagnosis of advanced renal cell carcinoma which was later confirmed by surgical excision and histology. Radical right nephrectomy, lymph nodes clearance, inferior vena cava cavatomy, and complete tumor thrombectomy were performed successfully. Perioperatively, he did not require cardiopulmonary bypass or deep hypothermic circulatory arrest. He had no recurrence during the follow-up period for more than 2 years after surgery.

**Conclusions:**

Advanced extension of renal cell carcinoma can occur with no apparent symptoms and be detected incidentally. In rare circumstances, atypical presentation of renal cell carcinoma should be considered in a patient presenting with right atrial mass detected by echocardiography. Renal cell carcinoma with inferior vena cava and right atrium extension is a complex surgical challenge, but excellent results can be obtained with proper patient selection, meticulous surgical techniques, and close perioperative patient care.

**Electronic supplementary material:**

The online version of this article (doi:10.1186/s13256-016-0888-5) contains supplementary material, which is available to authorized users.

## Background

Renal cell carcinoma (RCC) is a potentially lethal cancer with aggressive behavior and it tends to metastasize. RCC may present atypically with rare metastatic sites [1, 2]. Intravascular tumor growth along the renal vein into the inferior vena cava (IVC) occurs in up to 15 % of all patients with RCC and further extension of the tumor reaching the right atrium (RA) will be found in approximately 1 % of all patients [3].

## Case presentation

In a routine clinic follow-up, a 64-year-old Indian male with a past history of coronary artery bypass graft (CABG) surgery, congestive heart failure, and diabetes mellitus complained of worsening shortness of breath for 2 weeks. He reported normal urination and had no fever or weight loss. He had no past history or family history of cancer. On examination, he was obese (BMI 38 kg/m^2^), his radial pulse was regular (95/minute), afebrile, and his blood pressure was 110/70 mmHg. Cardiovascular examinations revealed a mid-line sternotomy scar, displaced apex beat, and diminution of heart sounds with no murmur. Fine crepitations were heard in his lung bases bilaterally. His liver and spleen were not enlarged. The results of the remainder of his examinations were normal. His laboratory results were as follows: hemoglobin, 13 g/dl; leukocyte count, 7.4×10^9^/L; platelet count, 159×10^9^/L; serum creatinine, 90 μmol/L; alanine aminotransferase (ALT), 15 IU/L; and urinalysis revealed plenty of microscopic red blood cells. His chest X-ray showed cardiomegaly and his ECG showed sinus rhythm with nonspecific T inversion at lateral leads.

**Fig. 1:**
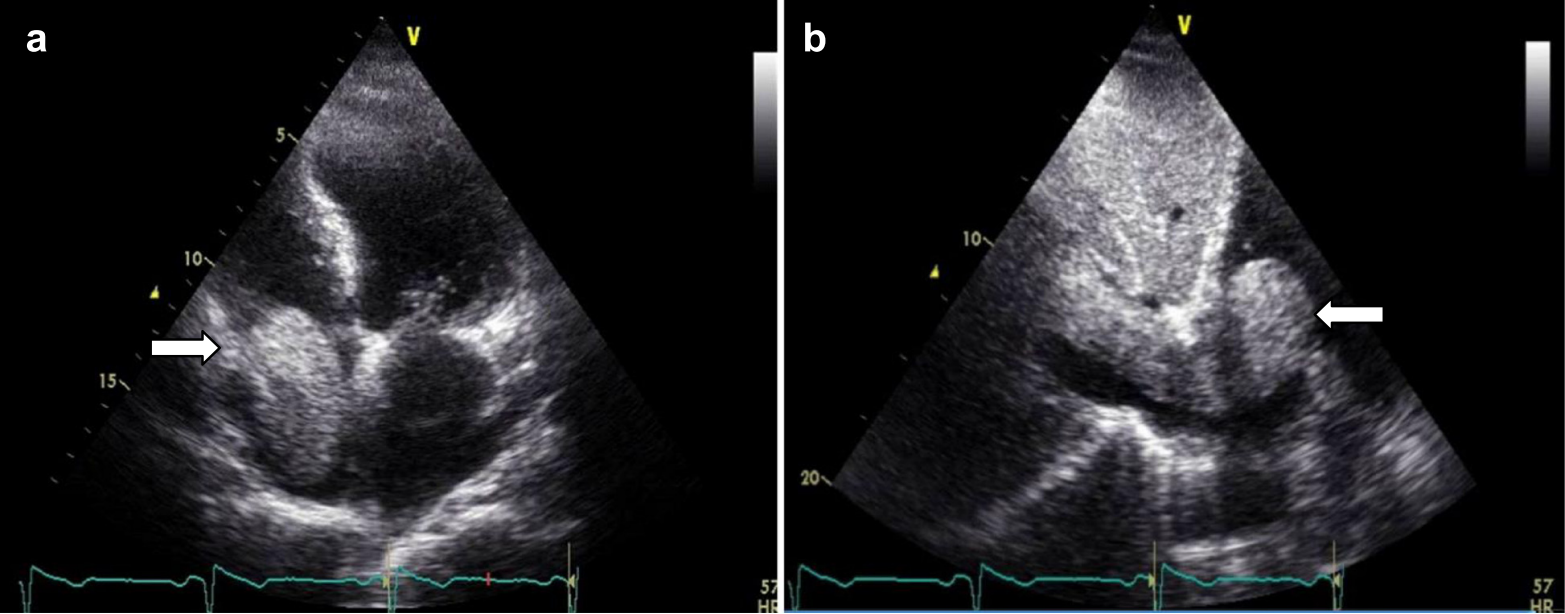
Apical four-chamber (**a**) and subcostal (**b**) views by transthoracic echocardiography showing the large mobile mass (*white arrow*) from inferior vena cava moving into right atrium and right ventricle

**Fig. 2:**
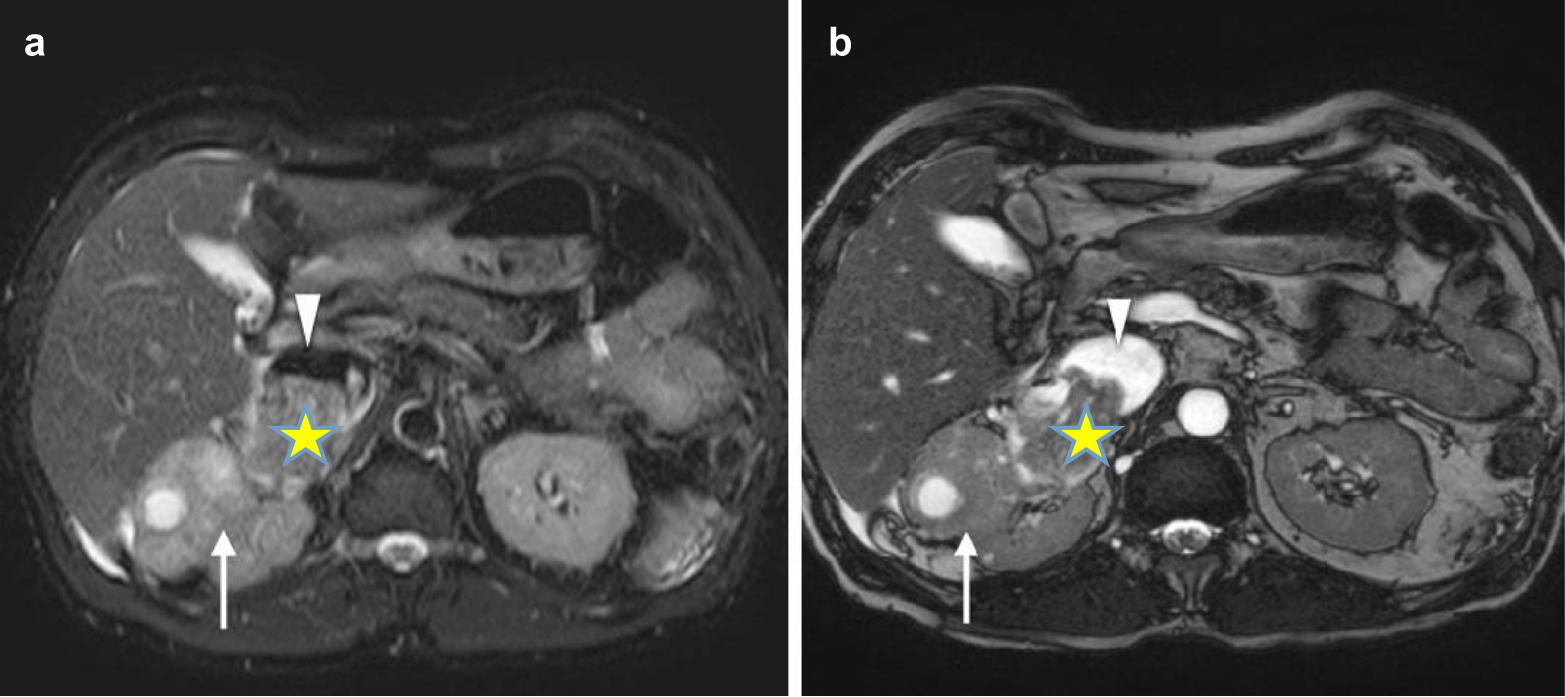
Axial T2-weighted fat saturation (**a**) and balanced turbo field echo (**b**) images showing the heterogenous right renal mass with cystic component (*arrow*) and formation of tumor thrombus in the dilated right renal vein (*yellow asterisk*) which protrudes into the intrahepatic inferior vena cava (*white triangle*)

**Fig. 3:**
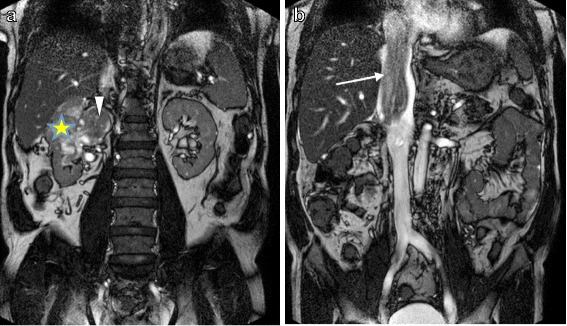
Coronal balanced turbo field echo images showing right renal mass (*yellow asterisk*) (**a**) with tumor thrombus in the engorged right renal vein (*white triangle*) extends cranially into the intrahepatic and suprahepatic inferior vena cava (*white arrow*) encroaching into the right atrium (**b**)

Transthoracic echocardiography (TTE) showed impaired left ventricular systolic function with ejection fraction of 40 %, and a large, highly mobile, “thumb-like” mass in the RA extending into right ventricle through the tricuspid valve with each systole (Fig. 1; see Additional files 1 and 2). His tricuspid valve function was normal. Abdomen magnetic resonance imaging (MRI) revealed a heterogenous lobulated mass (measuring 6.8×8.4×4.2 cm) in the upper and mid-pole of his right kidney suggestive of right RCC, with tumor thrombus extended into his IVC (infrahepatic, intrahepatic, and suprahepatic) and RA (Figs. 2 and 3). The imaging findings were consistent with a diagnosis of RCC level IV, classified according to the upper margin of the tumor in his IVC [4]. The diagnosis of RCC (clear cell type), Fuhrman grade 2, measuring 200×50×60 mm of total tumor size was confirmed later by histopathologic examination of the surgical specimens. Abdominal and thorax computed tomography (CT) was done for the purpose of tumor staging. Similar findings were found on CT scan with no evidence of distant metastasis. He was hospitalized for anticoagulation and heart failure therapy. Angiographic CABG conduits were assessed prior to surgery. Native coronary vessels were diffusely diseased. His left internal mammary artery graft and saphenous venous grafts were collectively normal. Subsequently, he underwent right nephrectomy, cavatomy, and thrombectomy successfully. The imaging findings were confirmed on surgical excision and histology. Findings from the nephrectomy specimen showed that the tumor was protruding from his renal vein and extended to the superior pole of his kidney. Grossly, the outer surface of his kidney was fairly smooth and lobulated and had not breached the renal capsule. Histopathologic examination showed the malignant cells were mainly clear cytoplasm with a distinct cell membrane; they exhibited fairly uniform, round, slightly irregular, vesicular nuclei with small nucleoli. The tumor infiltrated the renal parenchyma and extended into his renal capsule but did not invade his perirenal fat or Gerota’s fascia. The tumor extended into his renal pelvis and his dilated renal vein, and it adhered to the vessel wall. Finally, there was no evidence of recurrence (confirmed by repeat abdomen MRI and TTE) during the follow-up period for more than 2 years after surgery at our out-patient clinic.

## Discussion

**Fig. 4:**
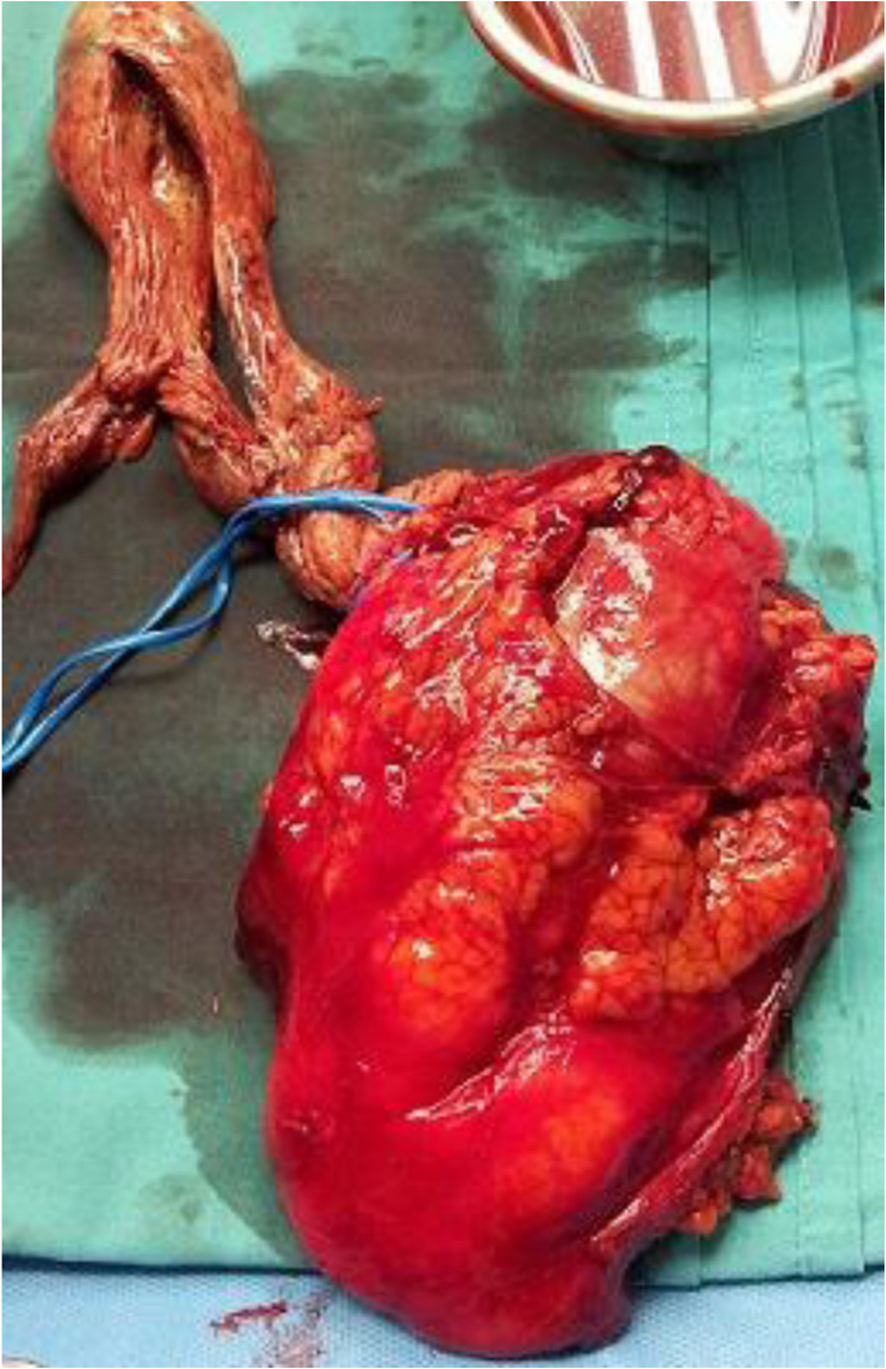
Surgically excised right renal tumor, cavatomy, and complete tumor thrombectomy

We reported a case of RCC with insidious presentation detected incidentally. The clinical course of our patient was subtle. He did not present with the classical triad of hematuria, flank pain, or flank mass, and had no apparent symptoms despite extension of the tumor thrombus into his IVC and RA. The diagnosis of RCC was considered after accidental detection of his RA mass by TTE. Common differential diagnoses of right atrial mass include thrombus (pulmonary emboli), cardiac tumors (primary or metastatic), and tricuspid valve vegetations, whereas uncommon differential diagnoses of right atrial mass include anatomic variants, coronary fistula, indwelling catheter, and pacer wires. With the more liberal use of radiological imaging techniques in current practice, the incidental finding of right atrial mass is particularly important for cardiologists, radiologists, or sonographers and the diagnosis of RCC should always be taken into consideration. A similar presentation of RCC extension into RA had been reported earlier in a series of case reports [3, 5, 6]. The majority (>70 %) of RCC are detected as incidental findings on imaging studies obtained for unrelated reasons [7]. At presentation, nearly 25 % of patients either have distant metastases or significant local-regional disease with no symptoms that can be attributed to renal cell carcinoma. One of the striking characteristics of RCC is its tendency to invade the renal vein, in which it may grow as a solid column of cells that extends up the IVC, sometimes as far as the right side of the heart [8]. Surgical treatment in patients with RCC extending into RA is challenging and controversy still exists regarding the safest strategy. The radical surgical strategy may include extracorporeal circulation with cardiopulmonary bypass (CPB) and sometimes deep hypothermic circulatory arrest (DHCA) [3]. Long-term survival is possible and the operative approach has been described earlier [4, 9]. For our patient, care was taken perioperatively since the surgical approach was complex and our patient had a past history of CABG. Fortunately, radical right nephrectomy, lymph nodes clearance, IVC cavatomy, and complete tumor thrombectomy (Fig. 4) were accomplished by a urologist in collaboration with a cardiothoracic surgeon. Although we considered CPB and DHCA in our preoperative plan, our patient did not require them. The reason was that he had a previous CABG which may have complicated the surgery. Furthermore, based on MRI findings the tumor thrombus in his IVC and RA did not adhere to adjacent structures. During surgical exploration, the surgeons managed to extract the tumor thrombus en bloc via the division of his right renal vein to his IVC. Histological analysis confirmed the diagnosis of clear cell type, the most common subtype of RCC, Fuhrman grade 2.

## Conclusions

Advanced extension of RCC can occur with no apparent symptoms and be detected incidentally. In rare circumstances, atypical presentation of RCC should be considered in a patient presenting with right atrial mass detected by echocardiography. RCC with IVC and RA extension is a complex surgical challenge, but excellent results can be obtained with proper patient selection, meticulous surgical techniques and close perioperative patient care.

## Consent

Written informed consent was obtained from the patient for publication of this case report and any accompanying images. A copy of the written consent is available for review by the Editor-in-Chief of this journal.

## Additional files

Additional file 1: Apical four chamber view by tranthoracic echocardiography. Renal cell carcinoma extending into ventricle with cardiac motion. (WMV 841 kb)

Additional file 2: Inferior vena cava view by transthoracic echocardiography. A "thumb-like" lesion protruding into right atrium and right ventricle. (WMV 833 kb)

## Abbreviations

CABG: coronary artery bypass graft
CPB: cardiopulmonary bypass
CT: computed tomography
DHCA: deep hypothermic circulatory arrest
IVC: inferior vena cava
MRI: magnetic resonance imaging
RA: right atrium
RCC: renal cell carcinoma
TTE: transthoracic echocardiography
